# No space left for intravenous thrombolysis in acute stroke: CONS

**DOI:** 10.1007/s11739-016-1448-0

**Published:** 2016-04-15

**Authors:** Keith Muir

**Affiliations:** Institute of Neuroscience and Psychology, University of Glasgow, Queen Elizabeth University Hospital, Glasgow, G51 4TF UK

**Keywords:** Thrombolysis, Recombinant tissue plasminogen activator, Stroke, Cerebrovascular disease, Thrombectomy, Endovascular treatment

## Abstract

Recent successful clinical trials of endovascular thrombectomy for large artery ischaemic stroke have established the value of this treatment modality as an adjunct to intravenous thrombolysis, not as an alternative: thrombectomy delivery was undertaken in the context of highly efficient networks for acute thrombolysis delivery and the great majority of patients received IV thrombolytic drug treatment. Even for the minority of acute stroke patients for whom thrombectomy is potentially relevant, access will be limited by geography and service infrastructure. Developments in intravenous thrombolysis in the near future will likely produce safer and more effective intravenous treatments. Intravenous thrombolysis will remain the first line of treatment for the great majority of acute stroke patients.

Intravenous thrombolysis (IVT) is an essential enabling technology for endovascular treatment, but current enthusiasm generated by the five endovascular treatment trials published in 2015 [1–5] should not distract from the wider reality of acute stroke treatment. For most patients, in most countries, and in most situations, IVT is the only proven and necessary therapy.

As indicated in the Pro argument, the great majority of endovascular treatment was adjunctive to IVT. In the published trials, only 15 % of patients were considered ineligible for IVT (and the protocols did not define what was accepted as “ineligible”—clinical practice has already moved far beyond the narrow licence criteria for alteplase, and provided solid evidence for the safety and effectiveness of doing so [6], and there is much subjectivity in determining “ineligibility.”) When the unpublished data from three further completed endovascular trials (THRACE, THERAPY and PISTE) are included, the proportion of “IVT ineligible” patients shrinks further, as all three trials required IVT for inclusion, and only 10 % had endovascular treatment in the absence of background IVT [7]. It is also potentially important that the endovascular trials were conducted in the setting of highly evolved and efficient networks of acute care, with the onset to treatment time of 85–127 min for starting IVT reflecting this, but also emphasizing how different this is from the average experience of acute stroke thrombolysis worldwide [8, 9]. It is known that thrombus properties change over time, and that clot composition may influence the effectiveness of endovascular therapy [10]. We do not yet know whether endovascular thrombectomy would be as effective in a situation where thrombolytic therapy had not been initiated at such early time points; clot may be intrinsically more resistant to removal as time passes. Thrombolytic drug treatment may modify clot composition favorably for endovascular treatment, but also might have beneficial effects on distal embolism and also on the microcirculation after removal of the main target clot, since alteplase causes prolonged hypofibrinogenaemia and hypoplasminogenaemia over many hours [11].

While the results of the SYNTHESIS expansion trial [12] are disregarded on grounds of older interventional technology being deployed (including intra-arterial thrombolysis in many cases), the absence of any benefit (and indeed trend towards poorer outcomes) in the intra-arterial treatment arm should give pause for thought. The median 1 h additional time taken for treatment initiation in the intra-arterial treatment arm of this trial seems likely to represent a major reason for failure. If time is brain, then initiating effective treatment at the earliest opportunity is both the ethical and appropriate course of action. Delaying for “better” treatment may be unnecessary and counter-productive.

It is often stated by enthusiasts for endovascular thrombectomy that IVT is “ineffective” for patients with large artery occlusion, yet evidence does not support this conclusion. In the IMS-3 trial, IVT alone achieved recanalization at 24 h in 80 % of proximal M1 occlusions, 86 % of distal M1 occlusions, and 28 % of ICA occlusions, despite a reduced dose of alteplase being used in a proportion of cases [13]. In addition, 21 % of those randomized to endovascular treatment and proceeding to angiography did not require treatment, in many cases because an initial proximal occlusion had partially or completely lysed, raising the concept of “futile transfer”—moving patients to endovascular centers (with potentially detrimental consequences for outcome) only to find that recanalization has already occurred. There is therefore no justification for assuming that endovascular treatment should be preferred over IVT in any situation where the patient is eligible for IVT, but rather that IVT should be initiated as quickly as possible and additional endovascular treatment delivered once treatment has commenced.

The proportion of patients for whom endovascular treatment may be suitable is unclear, with estimates based on trial entry criteria and registries hovering around 5 % of hospitalized acute ischaemic stroke patients. In the EXTEND-IA trial, the only one to report a screening log, 1 % of all hospitalized acute ischaemic stroke patients and 7 % of those eligible for IVT were entered into the trial [5]. It seems unlikely that endovascular therapy will be relevant to more than a small minority of patients even in advanced healthcare systems and restricted to those patients enjoying geographical access to appropriate treatment facilities. For most of the world, including some wealthy countries, interventional neuroradiology facilities and expertise are a scarce resource, and the development of necessary infrastructure will be both expensive and require time.

Finally, we assume incorrectly that IVT is a fixed entity. The stroke research community has collectively failed to address questions about the optimal agent and dosing regime for IVT, questions that cardiologists in contrast answered rapidly after initial proof of efficacy for IVT in myocardial infarction. The standard regime for IVT in stroke remains that tested in the NINDS trial reported in 1995 [14]. Most trials testing alternative (and pharmacologically superior) thrombolytic agents have tried simultaneously to expand the treatment time window or eligible patient groups, without success [15]. Only recently have investigator-led efforts to test newer thrombolytic agents against alteplase, or to evaluate different dosing regimes of alteplase, begun. Tenecteplase achieves superior recanalization, reperfusion and clinical outcomes in small clinical trials and with a better safety profile [16, 17]. Larger trials (ATTEST-2, TASTE, NOR-TEST) are underway to establish its effects [18]. Lower doses of alteplase might be as effective, but safer, and are under investigation in ENCHANTED. Combinations of thrombolytic drugs with antiplatelet agents or non-invasive physical approaches to improve clot penetration are also under investigation. Endovascular devices have evolved significantly over the course of a few years, and the older devices have proved to be inferior to the current generation [19, 20]. We should assume that IVT will evolve in a similar direction.

Better and faster reperfusion is the goal for ischaemic stroke care, and IV thrombolytic therapy will remain the base technology for the great majority of patients. Endovascular treatment is undoubtedly a great advance for the few who require it, and are able to access treatment within the necessary timescale, but it is a construction built on foundations of systems to deliver IVT. Perhaps the future will identify patients for whom a “direct to thrombectomy” pathway is better: but for the present this pathway lacks evidence and should not be the default, even in the small number of centers where it might conceivably be provided.
